# Exploring Trait Trade-Offs for Fungal Decomposers in a Southern California Grassland

**DOI:** 10.3389/fmicb.2021.655987

**Published:** 2021-04-20

**Authors:** Charlotte J. Alster, Steven D. Allison, Sydney I. Glassman, Adam C. Martiny, Kathleen K. Treseder

**Affiliations:** ^1^Ecology and Evolutionary Biology, University of California Irvine, Irvine, CA, United States; ^2^Department of Earth System Science, University of California Irvine, Irvine, CA, United States; ^3^Department of Microbiology and Plant Pathology, University of California, Riverside, CA, United States

**Keywords:** fungal traits, extracellular enzymes, drought, YAS framework, litter decomposition

## Abstract

Fungi are important decomposers in terrestrial ecosystems, so their responses to climate change might influence carbon (C) and nitrogen (N) dynamics. We investigated whether growth and activity of fungi under drought conditions were structured by trade-offs among traits in 15 fungal isolates from a Mediterranean Southern California grassland. We inoculated fungi onto sterilized litter that was incubated at three moisture levels (4, 27, and 50% water holding capacity, WHC). For each isolate, we characterized traits that described three potential lifestyles within the newly proposed “YAS” framework: growth yield, resource acquisition, and stress tolerance. Specifically, we measured fungal hyphal length per unit litter decomposition for growth yield; the potential activities of the extracellular enzymes cellobiohydrolase (CBH), β-glucosidase (BG), β-xylosidase (BX), and N-acetyl-β-D-glucosaminidase (NAG) for resource acquisition; and ability to grow in drought vs. higher moisture levels for drought stress tolerance. Although, we had hypothesized that evolutionary and physiological trade-offs would elicit negative relationships among traits, we found no supporting evidence for this hypothesis. Across isolates, growth yield, drought stress tolerance, and extracellular enzyme activities were not significantly related to each other. Thus, it is possible that drought-induced shifts in fungal community composition may not necessarily lead to changes in fungal biomass or decomposer ability in this arid grassland.

## Introduction

We are just beginning to understand how fungal growth and activity might shift under climate change (Todd-Brown et al., 2014; Xiao et al., 2018; Maynard et al., 2019). Fungal communities may transform to favor certain groups more adapted to these new environmental conditions (Kivlin and Treseder, 2014; Boddy et al., 2016). However, which fungal groups will be proliferating is still unknown. This issue is important in predicting decomposition, and ultimately, soil carbon (C) storage (Wieder et al., 2013). Specifically in the southwestern United States, which is experiencing major changes in precipitation patterns (Seager et al., 2007; Gibson et al., 2020), understanding how fungi will respond to drought is critical. One way to predict fungal response to drought stress is to investigate trade-offs among fungal traits.

Evolutionary and physiological trade-offs among traits may structure microbial communities and their contributions to ecosystem function (Ho et al., 2013; Malik et al., 2020b). Thus, examining trade-offs among fungal traits may be a useful approach in which to assess fungal response to drought. For example, under stressful conditions like drought, allocation of finite resources within organisms might require investment in one function at the expense of another function (e.g., production of compounds to combat stress at the cost of deferred growth; Bennett and Lenski, 2007). Here, we determined what trade-offs exist among traits of decomposer fungi in a Southern California grassland. Knowledge of these trade-offs may improve parameterization of trait-based microbial decomposition models as well as understanding of fungal physiology in this changing ecosystem.

We examined traits associated with three fungal strategies: growth yield, resource acquisition, and stress tolerance. Potential trade-offs between these life history strategies are described in Malik et al. (2020b) as a microbial modification to Grime’s competitor-stress tolerator-ruderal framework. In this newly proposed “YAS” framework (for yield-acquisition-stress tolerance), they hypothesize that microbes trade off resource investments among high yield (microbial biomass per unit of resource consumed), resource acquisition, or stress tolerance traits. Fungi with *high yield* strategies favor investment in central metabolism and assimilatory pathways necessary for building cellular components (Malik et al., 2020b). In contrast, fungi that allocate energy toward *resource acquisition* produce extracellular enzymes to break down various compounds in their environment. Investment in *stress tolerance* might involve increased production of osmolytes or polysaccharides to protect against desiccation (Schimel et al., 2007) and more effort spent on maintenance and repair of cellular structures (Kultz, 2005; Fuchs and Mylonakis, 2009).

These trade-offs are predicted in theoretical models (Pfeiffer et al., 2001; Allison, 2014; Manzoni et al., 2014). Empirical evidence for these trade-offs is strong for some, but limited for others. Several studies document trade-offs between growth yield and stress tolerance (Sterne and McCarver, 1978; Killham and Firestone, 1984; Dijksterhuis and de Vries, 2006; Gasch, 2007; Tiemann and Billings, 2011; Crowther et al., 2014; Garcia et al., 2020; Malik et al., 2020a). In contrast, trade-offs between resource acquisition and the other traits are less commonly studied. However, there is some support for trade-offs between stress tolerance and extracellular enzyme production, particularly for yeast (Treseder and Lennon, 2015; Morrison et al., 2018; Romero-Olivares et al., 2019). Soil microbial communities also exhibit trade-offs between C use efficiency, which could be a proxy for growth yield, and extracellular enzyme activity, especially for C-associated enzyme activity (Malik et al., 2019). Similarly, there are a few examples of trade-offs between growth rate and resource acquisition for fungal and bacterial species (Rinkes et al., 2011; Ramin and Allison, 2019; Whitney, 2019). Connecting trade-offs among these traits may be useful in predicting fungal response to climate change in the southwestern United States.

We hypothesized that fungal taxa from Southern California grassland preferentially invest in either growth yield, stress tolerance, or resource acquisition at the expense of the other traits, resulting in negative relationships between traits. To test this hypothesis, we measured growth yield (as fungal hyphal length per unit litter mass loss), drought stress tolerance (growth in drought vs. higher moisture levels), and resource acquisition (potential activity of four extracellular enzymes) traits from 15 decomposer fungi. Specifically, we investigated trade-offs between these traits by comparing their relationships across taxa.

## Materials and Methods

### Experimental Set-Up

Microcosms were established with grassland litter and fungal isolates. We collected dry, standing litter from Loma Ridge National Landmark in Southern California (33° 44' 13.2'' N, 117° 42' 42.0'' W, 365 m elevation), which is located on the traditional territory of the Acjachemen and Kizh communities (KIZH Nation, n.d.; Haas, 1995). This Mediterranean grassland is dominated by exotic and native annual grasses and forbs, including *Avena*, *Bromus*, *Lolium*, *Erodium*, and *Lupinus*, and native perennial grass *Nassella pulchra* (Potts et al., 2012; Martiny et al., 2016). The study site has an annual mean temperature of 17°C and mean precipitation of 30 cm (Kimball et al., 2014).

We roughly ground the litter (~1–2 cm) with coffee grinders and placed 5 g into 120 ml, amber widemouthed jars (Thermo Scientific; Supplementary Figure S1), then autoclaved them for 90 min at 121°C. Next, we inoculated the jars with one of 15 fungal strains that were previously isolated from Loma Ridge in 2017. These fungal strains represented 12 different species (some of the strains were the same species) from both Ascomycota and Basidiomycota phyla (Supplementary Table S1). The strains were identified *via* Sanger Sequencing using the ITS1F/ITS4 primer sets (White et al., 1990; Gardes and Bruns, 1993) and query coverage was at least 96% for all isolates according to the BLAST results. Isolates were stored as plugs in sterile water at room temperature for approximately 6 months (Jones et al., 1991) before regrowing on potato dextrose agar (with ampicillin and gentamicin) for the experiment. We transferred the regrown fungi to potato dextrose broth (with ampicillin and gentamicin) with continuous shaking. After 1 week, we centrifuged and rinsed the hyphae. The hyphal pellet was broken up and diluted with sterile tap water (optical density of 0.2 ± 0.02). We added 0.5 ml of dilute fungal hyphae to each jar of sterilized litter.

After inoculation, we added sterile deionized water to each microcosm so that the percent water holding capacity (WHC) equaled 4, 27, or 50%, and then placed the jars in an incubator set at 20°C. As a reference, we added ~0.05 ml to reach 4% WHC and ~17 ml of water to reach 50% WHC to the 5 g of litter. These moisture levels spanned conditions in the field site (Parolari et al., 2015) and aimed to capture the limits of water stress in this system. Additionally, from a practical sense, this was the minimum dryness level that was possible after the addition of the dilute hyphae. Each isolate (plus an additional uninoculated control) × moisture combination was replicated twice, for a total of 96 microcosms (15 isolates and 1 control × 3 moisture levels × 2 replicates = 96 microcosms). We incubated the microcosms (sterile litter, fungi, and water) for 5 weeks, airing the jars out weekly in a laminar flow hood for approximately 1 min to allow for oxygenation, while minimizing water loss and contamination risk. After the 5 week incubation period, fungal hyphae had spread throughout the microcosm litter. At that time, we froze a subset of the litter at −20°C for fungal hyphal biomass measurements and the rest at −80°C for extracellular enzyme activity measurements.

### Trait Measurements: Growth Yield

We measured fungal hyphal length and mass loss to represent the growth yield lifestyle “Y” lifestyle, sensu (Malik et al., 2020b). We calculated fungal hyphal length by staining and microscopy, using a procedure modified from Allison et al. (2013). In brief, 0.5 g of frozen litter was stirred in a 39.5 g/L sodium hexametaphosphate solution to extract the fungal hyphae. Subsamples (5 ml) of this solution were vacuum-pumped through a 0.2 μm nylon filter and stained with acid fuchsin. This was repeated twice for each sample. We mounted the filters on slides and dried them overnight at 60°C in a drying oven. Using an Axioplan 2 imaging microscope, we took five photos at random of each filter (10 photos total per sample) to be representative of the whole filter paper. We measured fungal hyphal length using AxioVision and calculated fungal hyphal length per gram litter using the method described in Shen et al. (2016). Mass loss was determined by calculating the difference in litter mass at the beginning and end of the 5 week incubation. To estimate growth yield, we divided fungal hyphal length (m per microcosm) by total litter mass loss from the microcosm.

### Trait Measurements: Resource Acquisition

We measured the potential extracellular enzyme activity of four enzymes involved in decomposition to assess resource acquisition traits (“A” lifestyle, sensu, Malik et al., 2020b). The enzymes measured included cellobiohydrolase (CBH) and β-glucosidase (BG) to estimate potential for cellulose degradation, β-xylosidase (BX) to estimate potential for hemicellulose degradation, and N-acetyl-β-D-glucosaminidase (NAG) to estimate potential for chitin degradation. We chose these enzymes because they are highly relevant in degrading plant fibers (Sinsabaugh et al., 2002) and fungal cell walls (Wohl and McArthur, 2001). We prepared sample homogenates and conducted fluorometric enzyme assays following methods described in Alster et al. (2013). In brief, 0.2 g of litter was added to 75 ml of 25 mM maleate buffer (pH 6.0) and homogenized using a Polytron automated homogenizer. About 200 μl of homogenate was added per well to 96-well plates with eight replicates per sample per assay. Each well also contained 50 μl of a fluorescent substrate. Controls of the sample homogenate, substrate solution, blanks, and standards were also replicated in eight wells. After 1 h, 10 μl of 1.0 M NaOH solution was added to each well to stop the reaction. Fluorescence was measured at 365 nm excitation and 450 nm emission. Enzyme activity was calculated according to Equation 1 in Alster et al. (2013) using the average values from the samples and controls. To control for enzyme activity being a function of fungal growth, we standardized potential extracellular enzyme activities per microcosm by multiplying by total litter mass remaining at the end of the experiment.

### Trait Measurements: Stress Tolerance

To quantify drought stress tolerance of the isolates (“S” lifestyle, sensu, Malik et al., 2020b), we characterized their response to the three moisture levels. We reasoned that if an isolate produced more hyphae under the driest treatment, compared with the wettest treatment, then that isolate was relatively stress tolerant. Conversely, if an isolate grew best at the wettest treatment, but declined under drier conditions, then that isolate was less stress tolerant. To capture these patterns, we calculated a moisture association index (MAI) that considered total fungal hyphal length in each microcosm at each moisture level:

$$\begin{array}{l} \mathrm{MAI}=\frac{\begin{array}{l} \left({\mathrm{Hyphal length}}_{4\%}\times 0.04\right)+\left({\mathrm{Hyphal length}}_{27\%}\times 0.27\right)\\ +\left({\mathrm{Hyphal length}}_{50\%}\times 0.50\right) \end{array}}{\begin{array}{l} {\mathrm{Hyphal length}}_{4\%}+{\mathrm{Hyphal length}}_{27\%}\\ +{\mathrm{Hyphal length}}_{50\%} \end{array}}\\ \;\;\;\;\;\;\;\;\;\;\;\;\;\;\;\;\;\;\;\times 100\% \end{array}$$

where Hyphal length*_4%_* was the hyphal length of the isolate in the 4% moisture treatment; Hyphal length*_27%_* was the hyphal length in the 27% moisture treatment; and so on. In theory, MAI could range between 4 (if the isolate only grew at 4% moisture) and 50% (if the isolate only grew at 50% moisture). The larger an isolate’s MAI, the less drought stress tolerant it was. We used this indicator because it weights the effect of moisture rather than treating growth at all moisture levels equally. This therefore “penalizes” fungi that have a much higher growth rate at higher moisture levels. While it is possible that this index could overestimate stress tolerance if growth peaked at the intermediate moisture level, or underestimate stress tolerance if growth was the same at all moisture levels, we generally did not find this to be the case for our isolates (Supplementary Figure S2).

### Statistical Analysis

First, we tested whether isolates differed in the traits related to the three lifestyles: high growth yield, resource acquisition, and stress tolerance. To determine whether isolates possessed different degrees of stress tolerance (S lifestyle), we asked whether they varied in their responses to moisture. We performed a fully factorial ANOVA, with growth yield as the dependent variable, and moisture treatment and isolate as independent variables. A significant interaction between moisture treatment and isolate would indicate that isolates differed in responses to moisture. In addition, a significant main effect of isolate on biomass would support differences among isolates in the Y lifestyle.

Next, we conducted a series of fully-factorial ANOVAs to assess whether isolates varied in extracellular enzyme activity (A lifestyle). Dependent variables were activities of CBH, BG, BX, or NAG. Independent variables were isolate and moisture level.

To test our hypothesis that fungal isolates would exhibit trade-offs, we checked for negative relationships between the Y, A, and S traits. Within each moisture treatment there were no non-linear relationships, so we used a linear model to test relationships between traits. Specifically, we conducted a series of hierarchical linear mixed regressions between pairs of traits, with moisture as a covariate. Because isolates that were more closely related to one another may have tended to display similar traits, we included phylum, class, order, and family as nested random factors. This approach is similar to applying a phylogenetic independent contrast (Ricklefs and Starck, 1996), while allowing us to account for the moisture covariate. Significant negative relationships between pairs of traits would support the hypothesis. Growth yield was ranked before this analysis, because those data were not normally distributed.

We also checked for relationships between each of the four extracellular enzyme activities and looked at how taxonomic variation affected each of the traits. We conducted hierarchical linear mixed regressions between pairs of enzymes, with moisture as a covariate and phylum, class, order, and family as nested random factors. We also ran hierarchical linear models, with each trait as the dependent variable; and phylum, class, order, and family as nested factors to determine the influence of taxonomy. In all cases, statistical tests were conducted with Systat 13 (SPSS, 2017).

Finally, as an additional measure to assess trade-offs among the traits simultaneously, we conducted a redundancy analysis (RDA) in R version 3.5.3 (R Core Team, 2019). We used the “vegan” package (Oksanen, 2020) to conduct the RDA with moisture and fungal species as factors and phylum, order, and family as a random hierarchy, and growth yield, hyphal length, mass loss, and the enzyme activities as the dependent variables. We conducted a permutation test, similar to an ANOVA, for the RDA to determine significance.

## Results

### Variation Among Fungal Isolates in Traits and Moisture Responses

**Figure 1:**
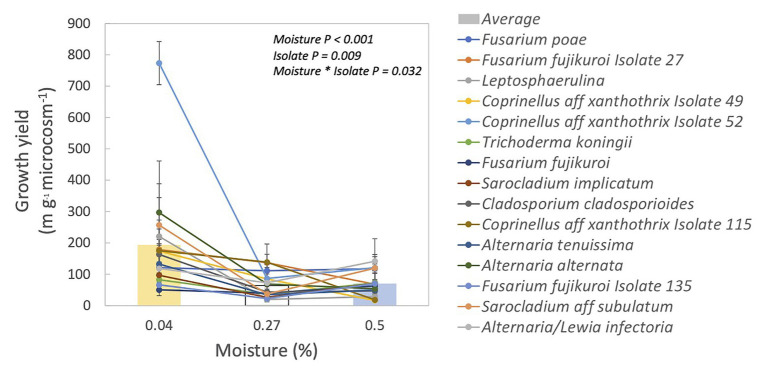
Growth yield for each isolate and their relationship to incubation moisture level. Each line represents one isolate. Symbols are means ± 1SE of two replicates. Bars are averages of all isolates.

Fungal isolates differed significantly in their responses to moisture, based on their growth yield at low vs. medium and high moisture levels (Table 1, moisture × isolate interaction: *p* = 0.032). Overall, average growth yield from all of the isolates peaked at the lowest moisture level (Figure 1). Isolates also varied in total hyphal length. Some isolates, like *Coprinellus* aff *xanthothrix* Isolate 52, appeared most abundant in the driest treatment; others, like *Fusarium poae*, appeared to peak in the wettest treatment (Supplementary Figure S2). Across isolates, the MAI ranged from 18 to 36%.

**Table 1:** Statistical results from fully-factorial ANOVA.

Trait	Moisture^†^	Isolate	Moisture × isolate
Growth yield	***F***_**2,45**_ = **24.222**, ***p*** d< **0.001**	***F***_**14,45**_ = **2.565**, ***p*** = **0.009**	***F***_**28,45**_ = **1.855**, ***p*** = **0.032**
Cellobiohydrolase activity^‡^	***F*_2,45_ = 7.815, *p*** = **0.001**	***F*_14,45_ = 2.614, *p*** = **0.007**	*F*_28,45_ = 1.359, *p* = 0.176
β-glucosidase activity	*F*_2,45_ = 2.085, *p* = 0.136	***F*_14,45_ = 1.949, *p* = 0.046**	*F*_28,45_ = 1.307, *p* = 0.208
β-xylosidase activity	*F*_2,45_ = 1.599, *p* = 0.213	*F*_14,45_ = 0.813, *p* = 0.651	*F*_28,45_ = 0.908, *p* = 0.600
N-acetyl-β-D-glucosaminidase activity	***F*_2,45_ = 5.666, *p* = 0.006**	*F*_14,45_ = 1.430, *p* = 0.179	*F*_28,45_ = 0.831, *p* = 0.695

† Dependent variable: trait value, independent variables: moisture and isolate. Significant p-values in bold.

‡ Cellobiohydrolase (CBH) digests cellulose;β-glucosidase (BG) digests cellulose; β-xylosidase (BX) digests hemicellulose; and N-acetyl-β-D-glucosaminidase (NAG) digests chitin.

In contrast, isolates did not vary significantly in the degree to which extracellular enzyme activity was sensitive to moisture (Table 1, moisture × isolate interaction: *p* > 0.176 for all enzymes). Although, across all moisture levels, isolates differed significantly from one another in CBH activity and BG activity (Supplementary Figure S3). In addition, across all isolates, growth yield, CBH activity, and NAG activity differed among moisture regimes. Specifically, CBH activity peaked at intermediate moisture, while NAG activity increased linearly with moisture.

Several traits also varied with taxonomy. MAI varied significantly at the phylum and family levels (Supplementary Table S2, *p* < 0.01). BG activity varied significantly at the family level (Supplementary Figure S2, *p* = 0.015), while NAG activity varied significantly at the class level (Supplementary Table S2, *p* = 0.047). Growth yield, CBH activity, and BX activity did not vary with taxonomic rank.

### Relationships Between Moisture Responses, Growth Yield, and Enzymes

**Figure 2:**
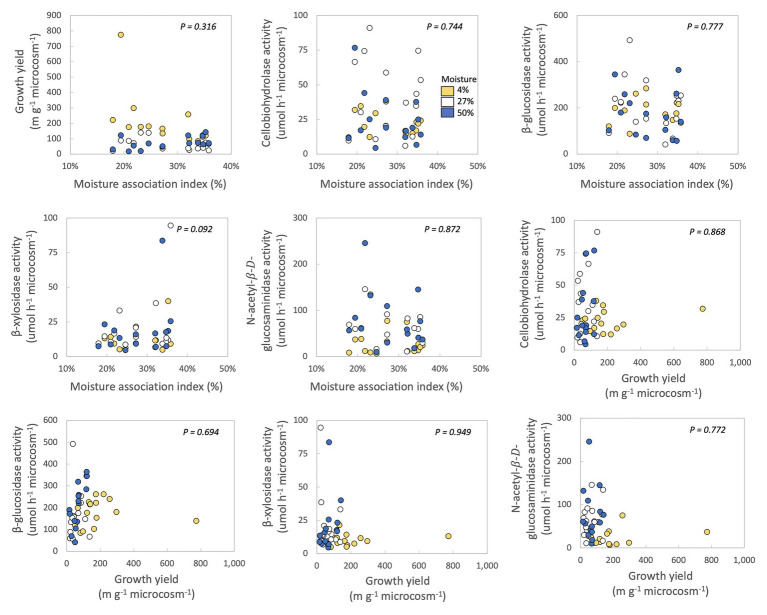
Correlations between moisture association index (MAI), growth yield, and extracellular enzyme activities of fungal isolates. Each point represents one fungal isolate (mean of two sampling replicates). Colors represent moisture level. *p*-values are for relationships between traits across all samples, with moisture as a covariate and phylum, class, order, family, and genus as nested random factors. Correlations between the ranked growth yield data and the other traits can be found in Supplementary Figure S4.

**Figure 3:**
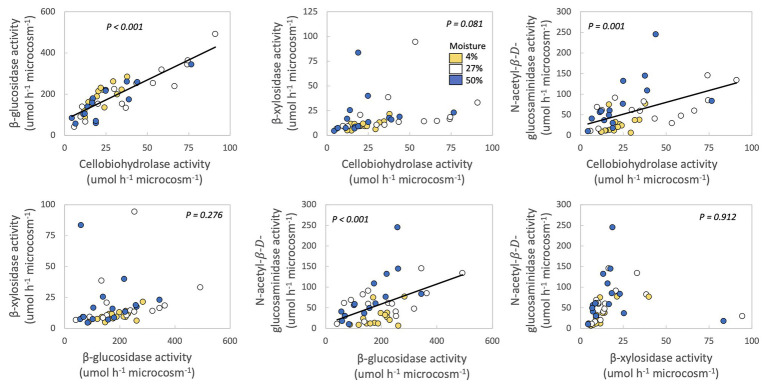
Relationships between extracellular enzyme activities of fungal isolates. Each point represents one fungal isolate (mean of two sampling replicates). Colors represent moisture level. *p*-values are for relationships between traits across all samples, with moisture as a covariate and phylum, class, order, family, and genus as nested random factors. Lines are best fit for significant relationships.

In no case were MAI or growth yield significantly related to any other trait we measured (Table 2; Figure 2). Thus, we rejected our hypothesis – these traits were not negatively related to one another. We found some support for a trade-off between growth yield and the extracellular enzyme activities based on the RDA (Supplementary Figure S5), however, this analysis was not significant (*p* = 0.561). Additionally, we also examined relationships between extracellular enzyme activities. CBH activity was significantly and positively related to BG and NAG (Table 2; Figure 3, *p* < 0.001, *R* = 0.875 for BG and *R* = 0.507 for NAG). β-glucosidase and NAG were also significantly positively related to one another (Figure 3; *p* < 0.001, *R* = 0.474).

**Table 2:** Statistical results examining pairwise relationships between fungal traits.

Traits^†^	Growth yield	Cellobiohydrolase activity	β-glucosidase activity	β-xylosidase activity	N-acetyl-𝛽-D-glucosaminidase activity
Moisture association index	*t* = −1.019, *p* = 0.316	*t* = 0.329, *p* = 0.744	*t* = 0.285, *p* = 0.777	*t* = 1.739, *p* = 0.092	*t* = 0.162, *p* = 0.872
Growth yield	--	*t* = 0.168, *p* = 0.868	*t* = 0.397, *p* = 0.694	*t* = 0.065, *p* = 0.949	*t* = −0.292, *p* = 0.772
Cellobiohydrolase activity		--	***t*** = **12.050, *p*** < **0.001**	*t* = 1.803, *p* = 0.081	***t*** = **3.571**, ***p*** = **0.001**
β-glucosidase activity			--	*t* = 1.109, *p* = 0.276	***t*** = **4.219**, ***p*** < **0.001**
β-xylosidase activity				--	*t* = −0.112, *p* = 0.912

† Hierarchical linear mixed regression, with moisture as covariate and phylum, class, order, family, and genus as nested random factors. Degrees of freedom = 31 in all cases. Significant *p*-values in bold.

## Discussion

In this study, we investigated if fungi from a Mediterranean grassland sorted according to the YAS framework (Malik et al., 2020b), in order to improve predictions of fungal response to climate change in southern California. We found minimal evidence for evolutionary or physiological trade-offs between traits associated with growth yield, resource acquisition, and stress tolerance. Specifically, there were no significant negative relationships among growth yield, drought stress tolerance, or activities of four extracellular enzymes that target cellulose-C, hemicellulose-C, and chitin-N (Figure 2). Perhaps investment in stress tolerance traits or extracellular enzyme production did not come at a noticeable cost to growth yield or to one another. Alternately, traits related to the YAS lifestyles may have all been important for maintaining fungal fitness in this ecosystem, so that fungi may prioritize investment in all of these traits at the cost of other traits not measured here. No matter the underlying mechanism, these results suggest that greater drought stress under climate change will not necessarily select for or against fungi with high growth yield or strong resource acquisition at this site.

For comparison, previous empirical studies at this field site have reported negative relationships between growth yield and stress tolerance, albeit in the whole microbial communities and bacterial isolates (Ramin and Allison, 2019; Malik et al., 2020a) as oppose to fungal isolates. In particular, the abundance of growth yield metabolites (ectoine and 5-oxo-proline abundances) are negatively related to stress tolerance metabolites (aspartic acid and adenosine) among whole microbial communities at this ecosystem (Malik et al., 2020a). One reason for this discrepancy may be the absence of competition from other microbes in our study (Anthony et al., 2020). If competition is driving fungal community dynamics, fungi may increase growth yield or resource acquisition to monopolize space and resources at the expense of stress tolerance (Ghoul and Mitri, 2016; Wood et al., 2018). Fungi could also produce toxins or other compounds to target competitors (Chao and Levin, 1981; Riley and Gordon, 1999) at the expense of growth yield or resource acquisition. It would be interesting to examine how these fungal isolates would fare with respect to trait trade-offs with the addition of competition. Yet, a lab-based study from this site also found a trade-off between fast growth rate and potential extracellular enzyme activity for bacterial isolates, even though isolates were grown in the absence of competition (Ramin and Allison, 2019). Thus, even if these fungal isolates did not appear to experience trade-offs between growth yield and resource acquisition, it seems that other members of the microbial community do.

Studies examining these relationships specifically using fungal isolates in other systems have also reported trade-offs between stress tolerance vs. growth, or between growth rate and resource acquisition (Gasch, 2007; Whitney, 2019). In a review of genomic expression in response to environmental stress for several strains of Ascomycota isolated from unspecified environments, stress resistance came at the expense of cellular growth (Gasch, 2007). Similarly, in a study of 10 saprotrophic fungal isolates from a temperate forest in Massachusetts, Whitney (2019) observed a negative relationship between resource acquisition and specific growth rate. These studies were conducted on liquid media or agar (instead of litter; Gasch, 2007; Whitney, 2019), which may partially explain our differing conclusions. However, perhaps trade-offs between these traits become significant under wetter or cooler conditions than are experienced by the fungi in our Southern Californian grassland (Wallenstein and Hall, 2012).

Fungal activity seemed limited at lower moisture levels because decomposition rates were slower (Supplementary Table S1). However, it remains possible that the moisture levels in our microcosms were not dry enough to cause sufficient drought stress despite large differences in litter water content (4–50% WHC). Nevertheless, there is evidence that these fungal strains are adapted to drought stress. We found that some isolates had their highest growth at the lowest moisture level (Supplementary Figure S2), and growth yield was largest in the lowest moisture level for nearly all of the fungal isolates (Figure 1). The higher growth yield at the lower moisture levels could be driven by greater hyphal growth to forage for resources (Jennings, 1987), compared to the higher moisture levels. Lower growth yield in the higher moisture levels could also be due to lower carbon use efficiency (i.e., more CO_2_ production; Manzoni et al., 2012b). Investigating these confounding factors may be warranted in future studies of fungal response to drought in this ecosystem.

In general, fungi tend to withstand drought better than bacteria (Yuste et al., 2011; De Vries et al., 2012; Manzoni et al., 2012a; Alster et al., 2013), due to their desiccation-resistant morphologies (Barnard et al., 2013; Treseder and Lennon, 2015) and more stable fungal networks (de Vries et al., 2018). In our study system, the ability of fungal isolates to maintain growth yield and extracellular enzyme production, even during drought, may allow fungi to maintain ecosystem-level decomposition rates where climate change increases drought severity. The knowledge gained in this study has implications for trait-based models of microbial decomposition, which often assume that trade-offs exist between growth yield and functions such as enzyme production (Allison, 2014; Wieder et al., 2014; Allison and Goulden, 2017). This assumption for soil fungi may require revisiting in this arid ecosystem. However, it is also possible that resource acquisition (e.g., by extracellular enzymes) may be used to support investment in drought tolerance or yield, especially in carbon-rich litter. For example, C gained from litter could be invested in C-rich polysaccharides to protect against desiccation. If this mechanism dominates, it could explain the lack of trade-offs observed in this study.

Production of extracellular cellulases (CBH and BG), hemicellulases (BX), and chitinases (NAG) were, in many cases, positively correlated with one another among fungal taxa (Figure 3). These results are supported by several other studies also noting positive relationships between potential activities of these extracellular enzymes (Keeler et al., 2009; Sinsabaugh et al., 2009; Waring et al., 2014; Talbot et al., 2015; Jian et al., 2016). These positive relationships may exist because multiple enzymes are needed to digest the available organic matter. For example, BG is needed to break down the products of CBH (Tsai et al., 2014). Given that these microcosms contained fungal isolates, instead of a community, the fungi would have had to produce both BG and CBH, as well as endocellulases, to hydrolyze cellulose into glucose (Tsai et al., 2014). The C-acquiring enzymes BG and CBH and N-acquiring NAG may also be produced synergistically in order to maintain stoichiometric ratios if C and N are co-limiting (Sinsabaugh et al., 2009; Waring et al., 2014).

In this study, we selected traits representative of fungal physiology with known consequences for soil decomposition dynamics (Treseder and Lennon, 2015; Malik et al., 2020b). Other traits, such as hyphal morphology, sporocarp production, spore size, or pathogenicity (Zanne et al., 2020), may elicit stronger trade-offs than those associated with the YAS lifestyles. Measurement of other extracellular enzymes, for example, leucine amino peptidase to capture peptide degradation (Talbot et al., 2015) or oxidative enzymes to capture lignin degradation (Alster et al., 2013), might also have elicited stronger trade-offs than what we observed here. A more targeted approach to measuring stress tolerance, for example, by measuring markers for synthesis of osmolyte production or biomolecular repair (Malik et al., 2020b), could also have yielded stronger negative relationships with the growth yield or resource acquisition traits. We acknowledge that this study examined a relatively small fungal collection with limited diversity, potentially biasing our results. Additionally, perhaps the ability to be cultured self-selects for a specific type of isolate, which could influence the observed trait responses. However, prevalence of Ascomycota among these strains is representative of the sequences dominating dried leaf litter from the Loma Ridge site (Matulich et al., 2015; Glassman et al., 2018). Regardless, it is difficult – if not impossible – with current technology to include here the full complement of fungal species in this ecosystem. Finally, we limit our interpretations to Southern California grasslands until trade-offs can be assessed in fungi from other dry ecosystems.

In conclusion, fungi isolated from this grassland exhibited no detectable trade-offs between growth yield, resource acquisition, and drought stress tolerance. Since the drought stress tolerance exhibited by a given fungal isolate was not significantly related to its ability to break down organic matter, we do not necessarily expect that shifts in fungal communities under drought would alter fungal decomposer ability. By including this information in trait-based ecosystem models, we may improve predictions of ecosystem function under climate change in Southern California.

## Data Availability Statement

The original contributions presented in the study are included in the article/Supplementary Material, further inquiries can be directed to the corresponding author.

## Author Contributions

KT, SA, and AM developed the original concept and obtained the funding. CA designed and planned the experiment with supervision from KT. SG isolated the fungi. CA performed the experiment. CA and KT analyzed the data with input from SA. CA wrote the original manuscript draft with significant involvement from KT. All authors contributed to the article and approved the submitted version.

### Conflict of Interest

The authors declare that the research was conducted in the absence of any commercial or financial relationships that could be construed as a potential conflict of interest.
