# Supplementary material for: Exploring Trait Trade-Offs for Fungal Decomposers in a Southern California Grassland
Source: Front Microbiol. 2021 Apr 20;12:655987. doi: 10.3389/fmicb.2021.655987 (PMC8118720; doi:10.3389/fmicb.2021.655987)
Supplement: Supplementary file 2 [file Data_Sheet_1.docx]

**Supplementary Information**

**Figure S1**. Photographs of the mesocosms (autoclaved litter, fungal isolate, and water) after five weeks of incubation including, (A) side view, (B) top view of a dry, low fungal growth mesocosm, and (C) top view of a wetter, high fungal growth mesocosm.

**Figure S2.** Responses of fungal isolates to incubation moisture level, based on fungal hyphal length. Each isolate’s moisture association index (MAI) is reported. Isolates with higher MAI were less drought-tolerant. Symbols are means ±1SE of two replicates.

**Figure S3.** Extracellular enzyme activities for each isolate and their relationship to incubation moisture level. Each line represents one isolate. Symbols are means ±1SE of two replicates. Bars are averages of all isolates.

**Figure S4**. Correlations between the moisture association index and extracellular enzyme activities with ranked growth yield of the fungal isolates. Each point represents one fungal isolate (mean of two sampling replicates). Colors represent moisture level.

**Figure S5**. Constrained ordination of the redundancy analysis where length and ordination of arrows indicate with strength and direction of differences in fungal species for each explanatory variable (i.e., growth yield, hyphal length, mass loss, and the enzyme activities). The colors of each species indicates the moisture level.
